# Human-landing rate, gonotrophic cycle length, survivorship, and public health importance of *Simulium erythrocephalum* in Zaragoza, northeastern Spain

**DOI:** 10.1186/s13071-017-2115-7

**Published:** 2017-04-08

**Authors:** Ignacio Ruiz-Arrondo, Javier A. Garza-Hernández, Filiberto Reyes-Villanueva, Javier Lucientes-Curdi, Mario A. Rodríguez-Pérez

**Affiliations:** 1 Departamento de Patología Animal, Facultad de Veterinaria, Universidad de Zaragoza, Zaragoza, Spain; 2 Centro de Rickettsiosis y Enfermedades Transmitidas por Artrópodos Vectores, Centro de Investigación Biomédica de La Rioja (CIBIR), La Rioja, Spain; 3 Instituto Politécnico Nacional, Centro de Biotecnología Genómica, Laboratorio de Biomedicina Molecular, Reynosa, Tamaulipas Mexico; 4 Laboratorio de Biología Molecular, Universidad Autónoma Agraria “Antonio Narro” Unidad Laguna, Coahuila, Mexico

**Keywords:** Blackfly, *Simulium erythrocephalum*, Human landing activity, Parous rate, Gonotrophic cycle, Survival rate, Host-seeking behavior

## Abstract

**Background:**

*Simulium* (*Boophthora*) *erythrocephalum* (De Geer, 1776) is one of the blackfly species responsible for major public health problems in Europe. Blackfly outbreaks of this species are becoming more frequent, threatening public health in Spain. In the present study, bionomic parameters of *S. erythrocephalum* in northeastern Spain were estimated.

**Methods:**

*Simulium erythrocephalum* was collected from May through June 2015 in Zaragoza, Spain, using the human-landing-collection (HLC) method. Daily pattern of total and parous landing activity was estimated, as was the gonotrophic cycle (GC) length and survivorship (S) rate, using time series analysis.

**Results:**

Host-seeking females of *S. erythrocephalum* showed a bimodal human-landing activity pattern, with a minor and major peak at dawn and dusk, respectively; there was a significant negative association between human daily landing rate and temperature (*P* = 0.003) and solar radiation (*P* < 0.001). Overall, a daily landing rate (DLR) of 34 lands/person/day was estimated. Series of sequential data analysis on parity showed the highest significant (*P* < 0.001) correlation indices (*r* = 0.45 and *r* = 0.39 for raw and filtered data) for a 2-day time lag, indicating that the GC length corresponded to 2 days. Daily survivorship and parity rate were 0.85 and 0.72, respectively.

**Conclusions:**

*Simulium erythrocephalum* was confirmed as a nuisance species in Zaragoza, using the HLC method for the first time in Spain. The data offer insights into the ecology of *S. erythrocephalum*, which can improve management strategies of this pest in Spain.

## Background

Blood-sucking blackflies (Diptera: Simuliidae) are important to medical and veterinary health. As vectors of disease agents (leucocytozoonosis, human onchocerciasis, mansonellosis, bovine onchocerciasis and the virus that causes vesicular stomatitis) [1], they also cause intolerable nuisances because of their abundance and habit of swarming and biting [1]. Blackflies introduce salivary molecules into the biting lesion, which are responsible for severe allergic reactions [2, 3]. In Spain, blackfly populations have expanded recently. The city of Zaragoza in northeastern Spain, with about 700,000 inhabitants, is the centre of an emerging public-health problem because of the abundance of blackflies near the rivers [4]. In the city of Zaragoza, the number of medical consultations due to arthropod bites increased by more than 200% during 2011 and 2012 (14,146 and 18,000, respectively), compared to previous years, 2009 and 2010 (4210 and 4512, respectively) (data from the Public Health Department, Government of Aragon). In spite of the numerous medical reports concerning bites could be caused by any arthropod (e.g. bees, wasps, mosquitoes, spiders), all entomological studies carried out during the outbreaks in 2011 and 2012 demonstrated that the aforementioned biting increment was due to the abundance of blackflies at the Ebro riverbanks of the city of Zaragoza [4, 5]. Of the 55 species known from Spain [6, 7], *S. erythrocephalum* is assumed to cause the majority nuisance of problems in the country because its immature stages are commonly found together with non-human biting species (subgenus *Wilhelmia*) in rivers near the areas of nuisance reports [4]. The current study is the first to use the human-landing-collection (HLC) technique for collecting blackflies in Spain.

Bionomics of blackflies, in particular, spatial and temporal population dynamics of parous host-seeking females, are well studied in primary vector species of onchocerciasis. This type of study was crucial in onchocerciasis control and elimination programmes in Latin America and Africa. Although *S. erythrocephalum* does not share a geographical distribution with endemic areas for *Onchocerca volvulus,* some laboratory studies have shown this species could potentially be a competent vector of *O. volvulus* [8]. In addition, this species has been incriminated as a natural vector of *O. lienalis* [9] and *O. gutturosa* [10] in cattle in Europe.

Blackfly control programmes require entomological studies to assess the magnitude of the problem and implement optimal strategies of monitoring, surveillance and control [4]. Recently, anthropophilic and zoophilic blackfly species triggered outbreaks in different regions in Spain [11], but no data was available on the factor(s) that may have caused the outbreaks or on the bionomics of the adults of *S. erythrocephalum*. Thus, we present an entomological study of the bionomics of *S. erythrocephalum.* The length of the gonotrophic cycle, using time series analysis, daily pattern of human-landing activity and its correlation with environmental parameters, daily survivorship and parity of *S. erythrocephalum* were studied in northeastern Spain. The bionomics of *S. erythrocephalum* are discussed in relation to its role as an emerging public-health nuisance in Spain.

## Methods

### Study area

**Fig. 1:**
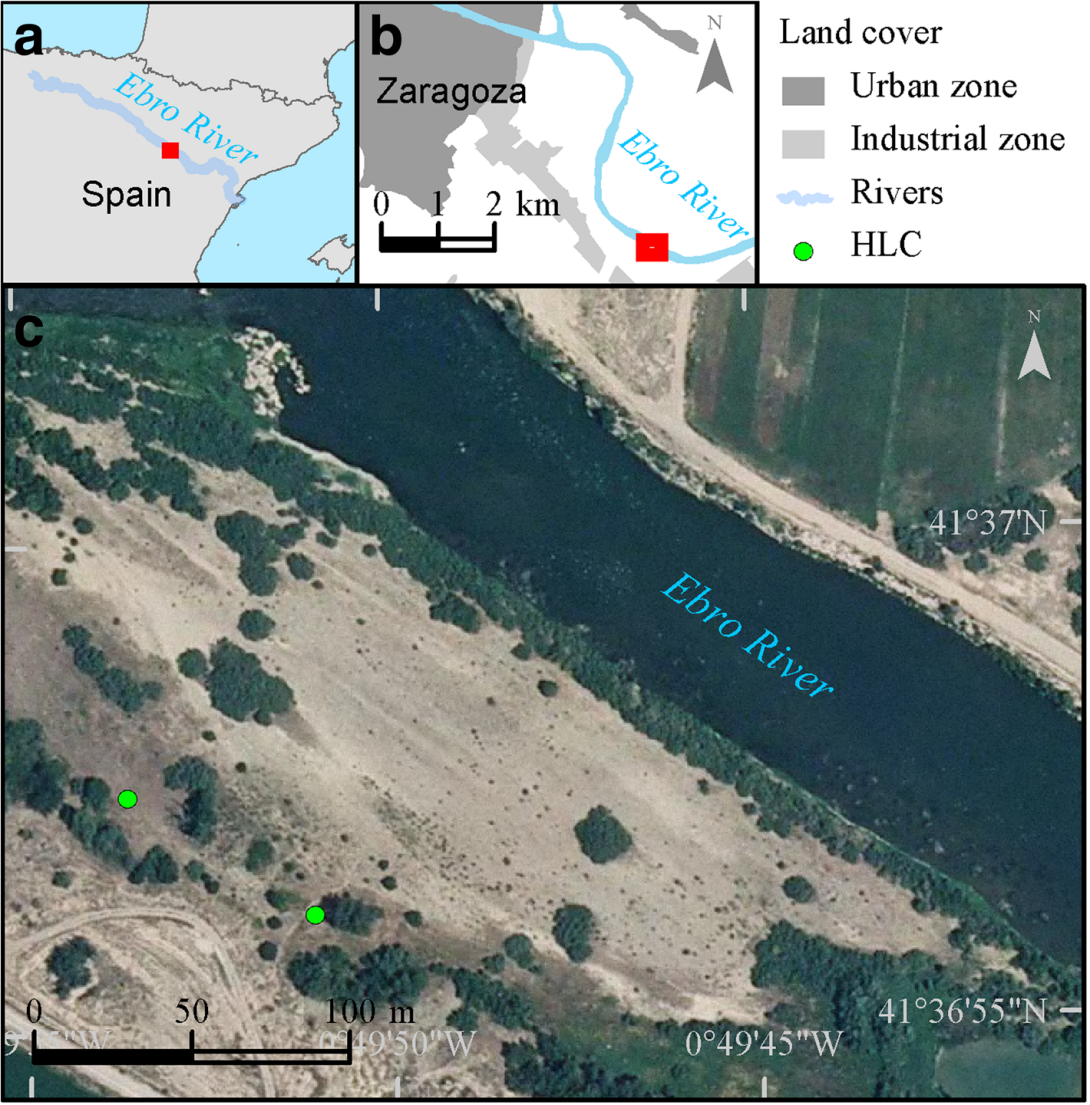
District of La Cartuja in the city of Zaragoza, Spain. In **a**, red square indicates the location of the city of Zaragoza in Spain. In **b**, red rectangle indicates the sampling site for host-seeking *S. erythrocephalum* females. In **c**, green circles indicate the position of the human-landing collectors (HLC)

The study was conducted in the district of “La Cartuja”, a rural suburb close to Zaragoza in northeastern Spain. The sampling plot was 115 m from the riverbanks of the Ebro River (41°36′55.97″N, 00°49′52.57″W, 185 metres above sea level, masl; Fig. 1c). It is a semi-naturalized area, 5.4 km downstream from a small dam that marks the end of the urbanised area of the city. The banks of the river are partially vegetated with typical species of riverside groves such as *Populus* spp. and *Salix* spp. and other shrub species such as *Rubus* spp*.* and *Tamarix* spp., and mainly surrounded by irrigated lands in the Valley Ebro, such as corn and lucerne*.*

In a preliminary survey conducted during May 2015, larvae, pupae, and adults of blackflies were collected along the Ebro River downstream from Zaragoza to select the best sampling plots to ensure collection of pre-imaginal stages and adults of *S. erythrocephalum*. Large larval and pupal populations of *S. erythrocephalum* have been documented recently in the area [4]. No larval control was performed during 2015 in this stretch of the river, which could have affected the present study.

### Daily pattern of human-landing activity of host-seeking *S. erythrocephalum*

Two collectors of flies (IRA and JAGH, served as volunteers) were situated 75 meters apart; both fly collectors were under the shadow of a tree to catch wild host-seeking females of *S. erythrocephalum* and other blackfly species (Fig. 1c). The fly collectors were not smokers and did not wear cologne or any lotion that could influence the host-seeking behavior of blackflies. The fly collectors wore dark short pants and light short-sleeved shirts and collected blackflies on exposed skin. Host-seeking females were captured while they were landing or attempting to feed on the human collector, and were placed in 75 × 12 mm plastic polystyrene tubes (Thermo Scientific, PA, USA) [12]. Tubes with flies were kept in Ziploc^®^ storage bags, maintained in gel-ice portable coolers during the field work, and transported to the laboratory where the flies were dissected to determine the gonotrophic status and the total number of nulliparous and parous flies. Sampling was carried out during the first 40 min of each hour starting at 06:30 and ending at 20:30 (Central European Summer Time - UTC + 2 h) for 12 consecutive days from May 29 through June 9, 2015. The collection period was selected to coincide with the sunrise (06:29) and sunset (21:38) at this latitude during the first day of the study in May. Sampling ended at 20:30 due to the absence of sunlight. Collectors were rotated daily between the two sampling sites to avoid position bias [13].

To determine the daily patterns of human-landing activity, the human-landing rate per hour was calculated as the total number of flies caught during each sampling period divided by the number of fly collectors, and it was expressed as the number of lands/person/hour. A proportion of blackflies land on the skin but they do not bite; therefore, the landing rate represents a maximum possible biting rate. A generalised linear mixed model (GLIMMIX) in SAS (SAS software version 9.4 13w18 Media) was used to fit the human-landing rate during the 12 days to a negative binomial distribution, and the least square means (LSM) of the sampling periods were compared. In addition, correlation and multiple linear regression analyses were performed to associate meteorological variables and sampling periods during the 12 days of the study.

### Meteorological variables

Weather variables such as temperature (°C) and relative humidity (%) were recorded using a digital weather station (EasyWeather™, Model PCE-FWS20, Albacete, Spain) that was placed under a tree at the sampling site. Wind speed (km/h), rainfall (mm^3^), and solar radiation (W/m^2^) were obtained from the nearest regional meteorological station in Pastriz (Zaragoza, Spain; 41°35′39.40″N, 00°43′49.48″W, 192 masl) 8.6 km from the sampling site. All weather variables were recorded daily and hourly from 06:00 to 22:00. Daily landing patterns depend on meteorological variables; hence, the data were normalised with the function Ln (x + 1), and a multiple linear regression was used to identify which variables were correlated with the daily landing pattern.

### Gonotrophic cycle length and survivorship rate of host-seeking *S. erythrocephalum*

The length of the gonotrophic cycle and survivorship rate of *S. erythrocephalum* were determined using an additional sampling of 10 consecutive days besides the 12 days of sampling for human-landing activity (i.e. June 10–19, 2015), making a total of 22 collection days for data analysis. However, sampling was carried out only from 18:30 to 20:30, which covered the main peak of daily human-landing activity. HLC was carried out as previously described. All specimens of *S. erythrocephalum* were dissected individually in a 0.85% saline solution and classified as nulliparous or parous based on absence or presence of sacculate dilatations and follicular relics in the ovarian tunic [14].

For data analysis, time series of 22 days were created. Each time series was constructed using the number of parous females (Pt) as the dependent variable (Y-axis) and the total number of females (Tf) as the independent variable (X-axis). Then, data were analysed by Mutero’s and Birley’s procedure [15], using the Auto Regressive Integrated Moving Average procedure (ARIMA procedure in SAS) to predict the length of the gonotrophic cycle in days. The r-coefficient for day 0 represents the correlation between Pt and Tf data pairs from flies captured the same day. The r-coefficient for a day was obtained by pairing daily Pt data with the corresponding Tf data of all previous days. It was assumed that a significant cross-correlation coefficient (r) between the time series expresses a time delay (u) equivalent to the length of the gonotrophic cycle. The highest significant cross-correlation coefficient (r) obtained after day zero (u = 0) indicated the number of days per gonotrophic cycle of wild populations of *S. erythrocephalum*. To avoid false peaks in the correlation coefficients caused by uncontrolled factors such as inconsistency in day-to-day collections or collections dominated by disturbances, the original data were transformed (filtration) by an auto-regressive equation with a time delay of 1 day of Z_T_ = X_T_ – β (Xt-1), where Z_T_ is the transformed data, X_T_ is the time series to be filtered (number of parous females collected in t day), Xt-1 is the total females collected before t day, and β is the estimated auto-regressive parameter (value of the slope of the linear regression applied to the data) [16]. Daily parity rate (DPR) was estimated as the ratio between ΣPf and ΣTf.

The survivorship or daily survival rate (DSR) was estimated by Davidson’s method [17], using the proportion of parous females in a population and calculated using the following equation:

$$ D S R = \sqrt[\  x]{DPR}, $$

where x is the length of the gonotrophic cycle in days. In addition, the survivorship rate per gonotrophic cycle was calculated by the cross-correlation method [15] employing the equation:

$$ \upsigma\ \left(\mathrm{x},\mathrm{y}\right)=\frac{{\displaystyle {\sum}_{i=1}^N}\left( x i - \kern0.5em \frac{\varSigma x}{N}\right)\left( y - \frac{\varSigma y}{N}\right)}{N=1}/{\sigma}_x=\frac{{\displaystyle \sum }{\left( x - \frac{\varSigma x}{N}\right)}^2}{N}, $$

where x is the total number of females and y is the number of parous females.

### Confirmation of gonotrophic cycle by oogenesis study

To confirm the length of the gonotrophic cycle, Christophers’s stages [18, 19] were recorded using 32 blood-engorged wild females of *S. erythrocephalum* fed by the two fly collectors. Flies were kept in the dark and maintained at 28 °C in plastic tubes, as described by Figueroa et al. [12]. Individual flies were dissected immediately after blood feeding at 6-h intervals for two consecutive days. Dissections of ovaries were made on glass slides containing a drop of 1× phosphate buffer solution (1× PBS). Ovaries were examined under phase-contrast microscopy. At least, three flies were examined for each period. Follicular development was characterised using Christophers’s stages as reported by Cupp & Collins [14].

## Results

### Daily pattern of human-landing activity

A total of 806 host-seeking females of *S. erythrocephalum* was caught. A DLR of 33.58 lands/person/day for *S. erythrocephalum* was estimated for Zaragoza. The highest daily landing rate was on May 29 (1st day) with 105.5 lands/person and the lowest on June 2 (5th day) with 4.5 lands/person.

The collection data (blackflies caught by two HLC) conformed to a negative binomial distribution and were thus examined using a general linear mixed model. The model used blackflies caught as the dependent variable and sampling day, fly collector, and sampling period as the independent variables. The model fits the data well (- 2 log likelihood value of 693.58 and a Pearson *χ* ^2^/degrees of freedom ratio of ~1 indicating no evidence for overdispersion). There was a significant association with sampling days (*P* < 0.001) and sampling period (*P* < 0.001). No evidence was found for an association of the two fly collectors with the number of catches (*P* = 0.815). Most lands were located on uncovered parts of the legs, but few on the arms.

**Fig. 2:**
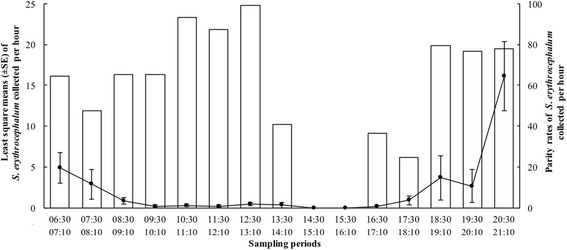
Bimodal daily landing activity pattern (line) and parity rates (bars) of host-seeking *S. erythrocephalum* females. For the Y_1_ axis, the values are expressed as the least square means ± standard error of the number of flies attracted by human-landing collectors, whereas for the Y_2_ axis, the values are the parity rates expressed as percentages

Human-landing activity of host-seeking females of *S. erythrocephalum* showed a bimodal pattern, with the first peak at sunrise between 06:30 and 08:10 (representing 23.1% of the lands) and the second peak between 18:30 and 21:10 (with 66.9% of the lands); the remaining landing activity occurred between 08:30 and 18:10 (10.0%) (Fig. 2). The parity rates of *S. erythrocephalum*, recorded during the first and second landing period, were 55.9% and 78.1%, respectively (Fig. 2). A high proportion (data not shown) of parous females, collected between 18:30 and 21:10, presented large sac-like follicular dilatations, indicating that oviposition had occurred within the previous 2–4 h [14].

### Effect of meteorological variables on the daily human-landing pattern (DHLP)

From all explanatory variables (day, hour, temperature, relative humidity, wind speed, rainfall, and solar radiation), the best model to estimate the DHLP included temperature (T) (*P* = 0.003) and solar radiation (SR) (*P* < 0.001). The multiple linear regression model was Ln (DHLP + 1) = 6.904–1.567 Ln (T + 1) - 0.224 Ln (SR + 1) (*R* ^*2*^ = 0.474, *P* < 0.001).

**Fig. 3:**
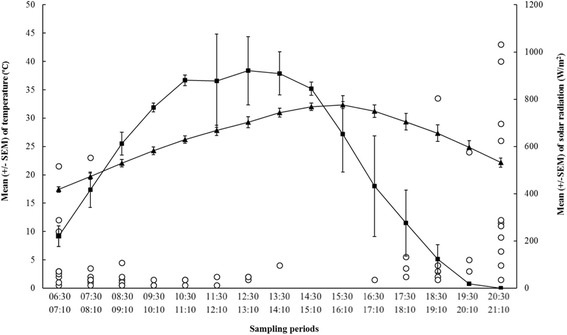
Relationship between the number of flies collected per hour of host-seeking *S. erythrocephalum* females and temperature and solar radiation. The white circles indicate the mean number of flies collected over 12 days. The black line with black triangles indicates the mean temperature (°C), and the black line with black squares indicates the solar radiation (W/m^2^)

The landing activity of *S. erythrocephalum* was negatively associated with increasing temperature and solar radiation (Fig. 3). Following the experiments on daily landing rate and GC and during the first week of July 2015, a heat wave in Spain increased the average temperatures. During this period, a mean of 32.15 °C was recorded during the second peak (18.30–20.30), 7.35 °C more than the usual average (24.8 °C) observed in the study. Blood-sucking females of *S. erythrocephalum* were not active at elevated temperatures; no specimens were caught by HLC.

### Estimation of gonotrophic cycle length and survivorship rate

A total of 875 females were collected, with a DPR of 0.72 (635 parous females). The highest capture occurred on day 2, with 128 females (86 parous females), and the lowest capture occurred on day 17, with only five females (4 parous females) (Table 1). The first significant (*P* < 0.05) correlation peak appeared on day 2, using filtered data. The cross-correlation coefficients for raw and filtered data were *r* = 0.45 and *r* = 0.39 for this time delay (u = 2), respectively. Using Davidson’s formula [17], we estimated the daily survival rate to be 0.85, using a daily parity rate of 0.72 (Table l).

**Table 1:** Number of captured *S. erythrocephalum* females in Zaragoza, Spain

Collection day	Total no. of females	No. of parous females	Daily parity rate
1	98	67	0.55
2	128	86	0.67
3	90	66	0.69
4	31	25	0.70
5	9	6	0.70
6	42	37	0.72
7	28	18	0.71
8	18	17	0.72
9	52	43	0.73
10	58	43	0.73
11	22	18	0.74
12	11	6	0.73
13	13	8	0.73
14	47	31	0.72
15	13	9	0.72
16	16	10	0.72
17	5	4	0.72
18	31	21	0.72
19	47	32	0.72
20	39	27	0.71
21	27	23	0.72
22	50	38	0.72
Totals	875	635	na

*Abbreviation*: na, not applicable

### Confirmation of gonotrophic cycle length by oogenesis

**Fig. 4:**
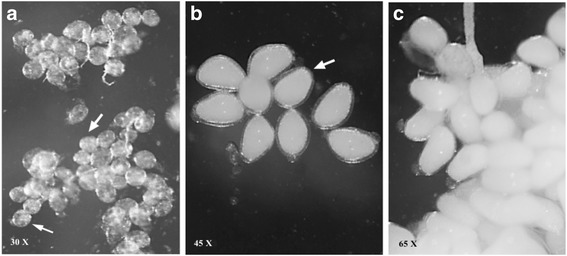
Oogenesis in *S. erythrocephalum*. **a** Six hours post-feeding: ovaries in stage III (arrows indicate that yolk fills about 30–40% of the follicular space). **b** 12 to 36 h post-feeding: ovaries in stage IV (yolk is present throughout the follicular space). **c** 42 to 48 h post-feeding: ovaries in stage V (mature ovarioles)

A general chronology of post-feeding changes in the ovary (Fig. 4) is described as follows: (i) 0 h: the blood meal occurred; (ii) 6 h post-feeding: the ovaries were in stage III, with the nucleus no longer visible and the yolk filling about 30–40% of the follicular space; the nurse cells were crowded in the distal portion of the follicle (Fig. 4a); (iii) 12 to 36 h post-feeding: the ovaries were in stage IV, namely, with yolk present throughout the follicular space and the nurse cells compacted distally (Fig 4b); and (iv) 42 to 48 h post-feeding: the ovaries were in stage V, with a true chorion and a visible micropyle (Fig. 4c), which indicated the end of oogenesis [14, 20].

## Discussion

The current study provides evidence that *S. erythrocephalum* could be a major responsible species for the blackfly nuisances in Zaragoza [5]. Larvae and pupae shared the breeding site with three other species, all in the subgenus *Wilhelmia*: *S. sergenti, S. lineatum,* and *S. equinum.* An association of *S. erythrocephalum* with *Wilhemia* spp. is common in other Spanish and European rivers [21, 22]. Species of *Wilhelmia* might be annoying for humans, as they fly about the faces of people, although humans are not their natural host. In Zaragoza, only *S. erythrocephalum* was captured using HLC. Similar biting habits have been reported including bites on the neck [23, 24].

The daily human-landing rate of 34 lands/person/day for *S. erythrocephalum* in Zaragoza indicates that this species is highly anthropophilic in Spain. Zivkovic [23] reported biting activity of *S. erythrocephalum* for all species of domestic animals. However, host preferences of *S. erythrocephalum* seem to differ, depending on the region of Europe. Cupina et al. [22, 24] indicated a tendency for this species to feed on humans over domestic animals. Jedkicka and Halgoš [25] reported a preference for horses over humans, and Crosskey [9] described this species as principally a pest of cattle and sometimes of humans.

The daily variation in landing rate has been observed in other studies [26] and may due to the existence of adult emergency waves from the breeding sites. The average of 34 lands/person/day is much higher than that classified as extreme risk (>11 *S. erythrocephalum* bites/person/afternoon) in Serbia [27]. During the first three days of our study, DLR was 106, 77 and 51 lands/person/day, above the mean DLR. The number of blackflies attacking their hosts can vary and is influenced by many factors [9]. In our study, the DLR could have increased in years when the Ebro River floods did not occur in winter and spring and, thus, did not pull up the macrophytes that provide a substrate for the preimaginal stages.

*Simulium erythrocephalum* showed a bimodal landing-activity pattern, with a minor peak at dawn and a major peak at dusk. Other European studies also have noted dusk as a period with a high number of bites by *S. erythrocephalum* [27, 28]. In Spain, the bimodal daily landing-activity pattern was affected by temperature and solar radiation.

With regard to solar radiation, flies showed host-seeking activity between 220.43 and 612.48 W/m^2^ in the morning, while most host-seeking activities were concentrated below 123.51 W/m^2^ in the evening. Host-seeking activity was greatest when the solar radiation was 0 W/m^2^ at 20:30 h, with the presence of a dim sunlight. Illumination is considered probably the most important external factor influencing the biting-activity pattern [9]. Flies showed host-seeking activity beyond the two peaks during cloudy periods after rain when temperature and solar radiation were within those ranges. The same behaviour was reported for *S. damnosum* (*s.l.*), which typically bites in the early morning and before dusk, but continues throughout the day during overcast weather [29, 30].

The optimal temperature for host-seeking activity of *S. erythrocephalum* was between 17.4 and 22 °C in the morning and between 22.17 and 27.35 °C in the evening. Our observations are similar to those reported by Berzina [31] within a temperature range of 15–27 °C in the Volga River Delta and 12–27 °C in the arctic. Females of *S. erythrocephalum* females were inactive when temperatures were above 29 °C, even though solar radiation remained in the optimal range for the second peak of host-seeking activity.

Dawn and dusk were the high-risk periods for suffering from biting by *S. erythrocephalum.* The optimal period for monitoring *S. erythrocephalum* is during the pre-sunset hours when solar radiation is below 123.51 W/m^2,^ and the temperature is 22.00–27.00 °C. Concentrating the efforts for adult sampling during this time can be less time and consuming and most cost effective if the evaluation of control strategies are to be implemented in Spain. HLC, along with adult trapping methods and larval monitoring at breeding sites, could be used routinely in future entomological studies in the country.

We did not evaluate the variation in the human-landing rates from one season to another. The study was carried out in late spring (May and June 2015) because very few *S. erythrocephalum* females were caught using HLC during previous months. During March and April, no adults of this species were caught using HLC. The first caught of host seeking *S. erythrocephalum* females was during the second week of May. May and June were the most suitable months for the sampling of *S. erythrocephalum* in this area. Bardin [32] pointed out that discomfort due to *S. erythrocephalum* species begins in May and increases during June and July in southern France. In September 2015, few *S. erythrocephalum* females (1–2 females were caught using HLC at dusk each day) were observed flying, and low numbers of larvae and pupae were found at the breeding sites. This decrease in population numbers of *S. erythrocephalum* during July, August, and September has been reported in other countries of Central and North Europe [24, 33–36]. Bardin [32] reported that *S. erythrocephalum* produced discomfort in France from spring through October. Thus, further studies are needed to define the spatial and temporal dynamics of *S. erythrocephalum* in Spain*.*

We observed no clear daily pattern of parity. Both peaks showed high parous rates, but parity was higher during the second peak at dusk (78%) than during the first one at dawn (56%). The catches of recent parous females, using the HLC method, indicated that *S. erythrocephalum* begin to search for and locate a new host almost immediately after oviposition, as reported for *S. ochraceum* (*s.l*.) [37]. The estimated gonotrophic cycle length of 2 days for *S. erythrocephalum* was shorter than those reported for certain species vectors for onchocerciasis: 3 days for *S. metallicum* (*s.l*.) [38], 4 days for *S. ochraceum* (*s.l*.) in Central America [26, 37] and about two and a half days for *S. damnosum* (*s.l*.) in West Africa [16, 39]. The 2-day gonotrophic cycle length was confirmed by oogenesis studies. However, Rubtsov [20] reported that the blood-meal to gravidity interval was 5–7 days for *S. erythrocephalum*, and Ham & Blanco [40] reported that it was 3–5 days at room temperature.

High parity rates of *S. erythrocephalum* indicate a high survivorship rate for this anthropophilic species. Survivorship rate in *S. erythrocephalum* was estimated to be 85%, with a daily parity rate of 72%, which is higher than that reported for other species, such as *S. ochraceum* (*s.l*.) (80%) in Mexico [26]. The same value of 0.85 elevated to the 2nd potency was 72.2%, the survival rate for the second day (first gonotrophic cycle).

High rates of adult survival coupled with the short gonotrophic cycle of *S. erythrocephalum* have important implications for public health. Females of *S. erythrocephalum* are potentially ready for human-host seeking 2 days after blood feeding and oviposition. This implies the possibility that many bites occur and that many eggs are laid at the breeding sites in a short period. *Simulium erythrocephalum* has a gonotrophic cycle shorter than other aggressive European species such as the *S. ornatum* complex or *S. lineatum*, which have blood-meal to gravidity intervals of 3–5 days [40, 41]. *Simulium erythrocephalum* could feed twice on humans, compared with once in those species with longer gonotrophic cycles. Short gonotrophic cycle and strong anthropophilic nature together with its efficiency for locating hosts, could lead *S. erythrocephalum* to emerge as an important human bite nuisance insect in Spain.

## Conclusions

Results indicated that *S. erytrocephalum* is an important anthropophilic blackfly collected by HLC method in the study region. The highest risk to suffer from *S. erythrocephalum* bites is during the pre-sunset hours. Human landing activity of host-seeking females of this species is greatly influenced by air temperature and solar radiation. A short gonotrophic cycle length and a high survival rate of *S. erythrocephalum* indicate a great success in the development of this species in Spain with the consequent impact on public health.

## Abbreviations

ARIMA: Auto regressive integrated moving average procedure
CEICA: Comité Ético de Investigación Clínica de Aragón
CG: Gonotrophic cycle
DHLP: Daily human-landing pattern
DLR: Daily landing rate
DPR: Daily parity rate
DSR: Daily survival rate
GLIMMIX: Generalised linear mixed model
HLC: Human landing collection
HLCP: Human landing collection periods
LSM: Least square means
PBS: Phosphate buffer solution
S: Survivorship
SR: Solar radiation
UTC: Coordinated universal time
